# A modified coupled partial differential equation method for synthetic aperture sonar interferogram denoising

**DOI:** 10.1016/j.isci.2026.115732

**Published:** 2026-04-14

**Authors:** Pan Huang, Yan Fan, Meng Wang, Lijie Guo

**Affiliations:** 1 School of Mathematics and Statistics, Weifang University, Weifang, Shandong, China; 2 College of Ecological Engineering, Shandong Ecology and Environment University, Weifang, Shandong, China

**Keywords:** earth sciences, oceanography, engineering, surface sensing

## Abstract

Interferometric synthetic aperture sonar (InSAS), a new high-resolution three-dimensional imaging device, is broadly utilized in underwater exploration. Interferogram noise removal is a necessary stage in InSAS image processing. The coupled partial differential equation (CPDE)-based method has been frequently used for image denoising. But the limitation of the traditional CPDE methods is that the edge strength function does not change over time, which leads to the convergence time becomes longer. This article puts forward a modified coupled partial differential equation (MCPDE) method for InSAS phase noise suppression that addresses the shortcoming of traditional CPDE methods. In the proposed method, a time-varying function is introduced to control the diffusion strength in edge regions. As the iterations progress, the function decreases, which guarantees the filtering intensity becomes weaken so that the detailed information will not be lost. Numerical simulations and real InSAS data experiments verified the performance of the proposed model.

## Highlights

• Propose a modified coupled partial differential equation
• Numerical solution of partial differential equations
• Analyze the convergence property of this method
• Obtained the denoised image

## Graphical abstract

**Figure:**
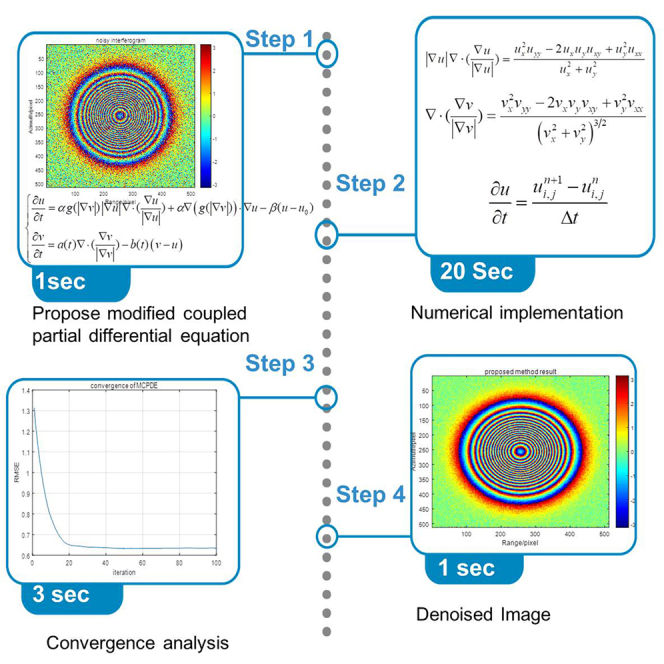

## Introduction

Recently, the exploration of ocean resources has created an urgent need for high-resolution imaging of underwater landforms and targets. Synthetic aperture sonar (SAS) is a high-resolution underwater imaging equipment that offers the advantage of azimuth resolution being independent of detection distance.^1^^,^^2^^,^^3^^,^^4^^,^^5^^,^^6^^,^^7^^,^^8^^,^^9^ Interferometric synthetic aperture sonar (InSAS), which utilizes two or more receivers based on SAS, can measure the height of underwater targets more accurately than SAS.^10^^,^^11^^,^^12^^,^^13^ Consequently, InSAS is extensively used in many fields, including seabed topography and geomorphologic mapping, marine engineering surveys, mineral resources development, underwater surveillance, underwater surveying, and engineering quantity assessment in channel dredging, underwater pipeline exploration, salvage of underwater sunker, archaeology, and the detection and identification of underwater targets.^14^^,^^15^^,^^16^^,^^17^^,^^18^^,^^19^^,^^20^^,^^21^^,^^22^ It is also employed in terrain-matching navigation.^23^^,^^24^^,^^25^

The signal processing steps of InSAS mainly include synthetic aperture imaging, complex image registration, interferogram generation, interferogram filtering, phase unwrapping, and digital elevation model (DEM) reconstruction.^26^^,^^27^^,^^28^^,^^29^^,^^30^^,^^31^^,^^32^^,^^33^^,^^34^^,^^35^^,^^36^^,^^37^^,^^38^^,^^39^^,^^40^^,^^41^^,^^42^^,^^43^ Because of the presence of noise, the interferogram generally cannot be used directly for phase unwrapping. To increase the signal-to-noise ratio (SNR) of the interferogram, reducing the influence of phase noise is urgently necessary in InSAS. This also benefits the reduction in difficulty of phase unwrapping and improvement in the accuracy of the DEM.

Among all interferogram denoising methods, the method based on partial differential equation (PDE) has been widely concerned due to its wonderful denoising performance and solid theoretical background. In addition, numerical algorithms for the PDE-based denoising model have been proposed. The PDE-based image denoising method aims to model the image using PDEs, with the PDE solution providing the desired denoised image. The advantages of the PDE denoising method include the following: it has the support of strong mathematical theories and incorporates traditional filtering methods into a new framework, making it more flexible than traditional filtering methods. Furthermore, it can be combined with compressed sensing and deep learning methods.

The primary PDE denoising method is the Perona-Malik (PM) model.^44^ It arrives the aim of edge preservation while eliminating noise, which depends on incorporating the gradient value of the image into the diffusion coefficient. The PM model can be formulated as follows:

(Equation 1) $$u_{t}=\mathrm{div}\left(c\left({\left|\nabla u\right|}^{2}\right)\nabla u\right)\text{,}$$

where *u* is the initial image before filtering, while ∇ and div represent the gradient and divergence operators, respectively. |∇*u*| is the gradient magnitude of *u*, $c\left({\left|\nabla u\right|}^{2}\right)=1/\left(1+{\left|\nabla u\right|}^{2}/\lambda^{2}\right)$ is the diffusion coefficient, and the gradient threshold *λ* is a positive constant. To avoid the shortcoming of the traditional spatial filtering methods that do not preserve the edge regions, the anisotropic diffusion method can simultaneously remove noise and preserve edges. This design performs well for additive noise but is ineffective for multiplicative noise. The PM model maintains image edges by controlling the diffusion strength using *c*(*s*^2^). The value of the diffusion coefficient *c* relies on the gradient of the image |∇*u*|. In the regions with small |∇*u*|, indicating relatively flat regions, *c* is large, leading to strong smoothing and pronounced filtering effects. Conversely, in the areas with big |∇*u*|, indicating rapid changes in image features, the small diffusion coefficient *c* diminishes the smoothing effect, making the filtering effect less pronounced. Peculiarly at the edges of the image, as the |∇*u*| is large enough, *c* approaches 0. Thus, diffusion is almost halted to preserve the edges.

The advantage of the PM model is that the diffusion coefficient can be automatically selected based on the gray change degree of the image (gradient modulus) without manually setting the filter window size, which is conducive to protecting the edge details. However, the PM model has the disadvantage of producing ill-posed solutions, which can be addressed through regularization.^45^ Many scholars have proposed regularized versions of the PM model, with the main regularization methods being spatial regularization and temporal regularization.^46^ The most influential spatial regularization PM (RPM) model, introduced by Alvarez in 1992, can be expressed as follows:

(Equation 2) $$u_{t}=\mathrm{div}\left(c\left({\left|\nabla u_{\sigma }\right|}^{2}\right)\nabla u\right)\text{,}$$

where *u*_*σ*_ = *G*_*σ*_∗*u* and *G*_*σ*_ is Gaussian kernel function with variance *σ*. The essence of *u*_*σ*_ is using the Gaussian kernel function to convolve with the original image *u*. That is, it performs Gaussian low-pass filtering on the original image *u*. Cottet and Germain utilized the reactive diffusion equation to implement regularization in the PM model.^47^ Weickert proposed two models for regularization: the edge-enhanced tensor diffusion model and the coherent enhanced tensor diffusion model.^48^ The temporal regularization method mainly includes transforming the equation into a system of equations with a time term. In addition, another important regularization method for the PM model involves solving its corresponding spatial numerical discrete form. As the spatial step size is relevant to the number of pixels, it is usually not required to converge the spatial step size to 0 (as in the continuous PM model form) to achieve regularization. Nordstrom proposed an anisotropic diffusion model with fidelity terms, which is equivalent to solving the steady-state format of the PM model and avoids the complexity of iteration time selection in the PM model. Gilboa proposed a forward and backward diffusion equation denoising model to achieve a better denoising effect by selecting special diffusion coefficients.^49^ Alvarez proved that every scale space that satisfies some basic structure, information reduction, and invariance axioms can be interpreted as a PDE model, and some characteristics of nonlinear diffusion denoising model can be well explained by the theory of scale space.^50^

The methods mentioned previously design the nonlinear diffusion equation denoising model from a diffusion perspective. Other denoising PDE models are derived using the variational method. The idea of variational denoising methods comes from measuring the statistical properties of noise. However, this problem is not well-posed; thus, adding regular terms can achieve a better solution. The primary formulation of the variational denoising method is as follows:

(Equation 3) minE(u)=R(u)+λF(f,u),

where *λ* > 0 is a constant, *R*(*u*) is the regularization term, and *F*(*f*,*u*) is the fidelity term. The design of the variational denoising model mainly focuses on improving the regularization and fidelity terms. Some variational models achieve noise removal by improving the fidelity term, such as maximum a posteriori estimation, which replaces the *L*^2^ fidelity term with the *L*^1^ fidelity term.^51^^,^^52^^,^^53^ The L^1^ norm is scale-invariant and effective for maintaining image contrast, but it is more complicated to calculate than the *L*^2^ norm. Huang performed an exponential transformation of the fidelity term in the literature, and Jin verified the uniqueness of the solution to the minimization problem in literature.^54^^,^^55^ Other variational models improve denoising by modifying the regularized term, including total variational regularization, fourth-order regularization, variable-exponential regularization, nonlocal regularization, and fractional-order regularization.^56^^,^^57^^,^^58^^,^^59^^,^^60^ Chen highlights that the noise removal ability of variational models can be improved by selecting different regularization and fidelity terms.^61^

The PDE denoising methods mentioned previously just consider the spatial regularization in the selection of the diffusion function and fidelity term. The results of numerical experiments indicate that the choice of diffusion function is crucial for obtaining a restored image. Thus, the choice should be made carefully. A major problem is that the diffusion functions mentioned above cannot inject past information into the iterative process of diffusion. To address this question, spatial-temporal regularization approaches were applied to noise removal, which is called the coupled PDE (CPDE) method.

The most commonly used CPDE model was proposed by Prasath, which can be expressed as follows:

(Equations 4 and 5) $$\left\{ \begin{array}{l} u_{t}=\mathrm{div}\left(c\left(v\right)\nabla u\right)\\ v_{t}=\lambda \mathrm{div}\left(\nabla v\right)+\left(1-\lambda \right)\left(\left|\nabla u\right|-v\right) \end{array}\right.$$

where 0≤*λ* ≤ 1 is a constant. This CPDE model is combined with the PM (1) and RPM model (2).^62^ The prototype of the PDE (4) is the PM model in (1). The diffusion coefficient function *c*(|∇*u*|) in (1) is separated into another variable *v* and incorporated into that function *c*(*v*). Notably, the gradient |∇*u*| acts like an edge map computed from the image *u* and is sensitive to noise, so it can result in a step effect. Therefore, this separation can achieve improved denoising as it allows for precise control of the edge map by a splitted PDE. The second PDE (5) in CPDE model plays an important role because it restricts the variable *v* to be like |∇*u*|. The parameter *λ* balances between the PM model (1) and the RPM model (2), which is crucial in localizing the denoising effects of the diffusion-based scheme. Specifically, higher presmoothing in the RPM model can lead to poor edge localization, while the PM model can lead to staircasing effects in flatten areas of the image. A balanced model can avoid these drawbacks and provide improved results. Some relevant CPDE models have been studied by researchers in the past.^63^^,^^64^^,^^65^^,^^66^

In Prasath model, the parameter *λ* is constant, it makes the strength of diffusion always invariant, which may lead to over-filtering. To address this shortcoming, the main contribution of this paper is that it introduces a time-variation function to control the strength of edge fidelity term, which ensures that the edges of the interferogram will not be overfiltered as time increasing.

## Results

**Figure 1:**
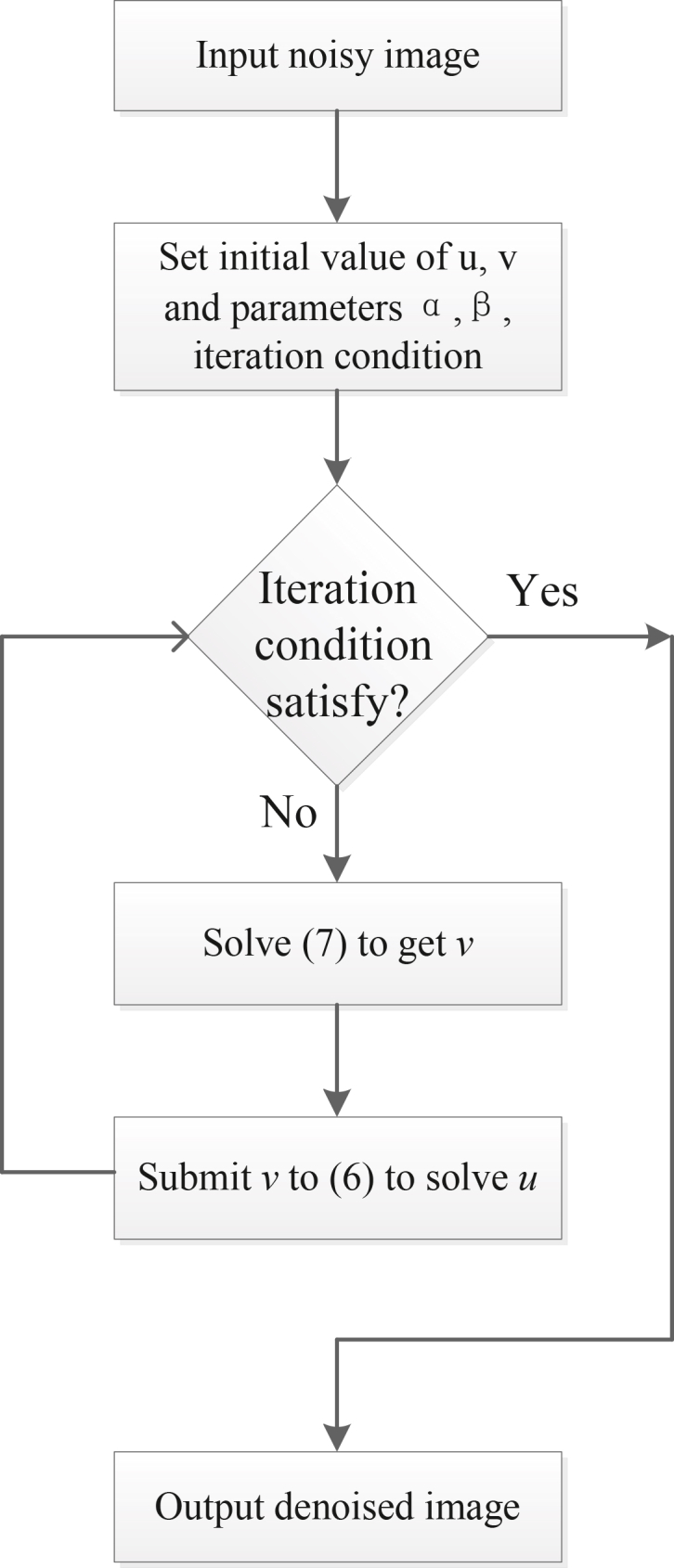
Flowchart of the presented MCPDE model

This section compares the proposed modified CPDE model (denoted as MCPDE) numerically with the RPM model, Prasath model and CPDE model in (denoted as CPDE).^66^ The solution flowchart of the MCPDE model can be seen in Figure 1. The simulation and real InSAS data experiments were conducted using MATLAB 2021a. The central processing unit used was an Intel Xeon Gold 128R with @2.1 GHz. The experiments are implemented without parallelization. The fixed parameters are given as *α* = 0.25, *β* = 0.05, *K* = 100, Δ*t* = 0.2.

### Numerical simulation experiments and result analysis

**Figure 2:**
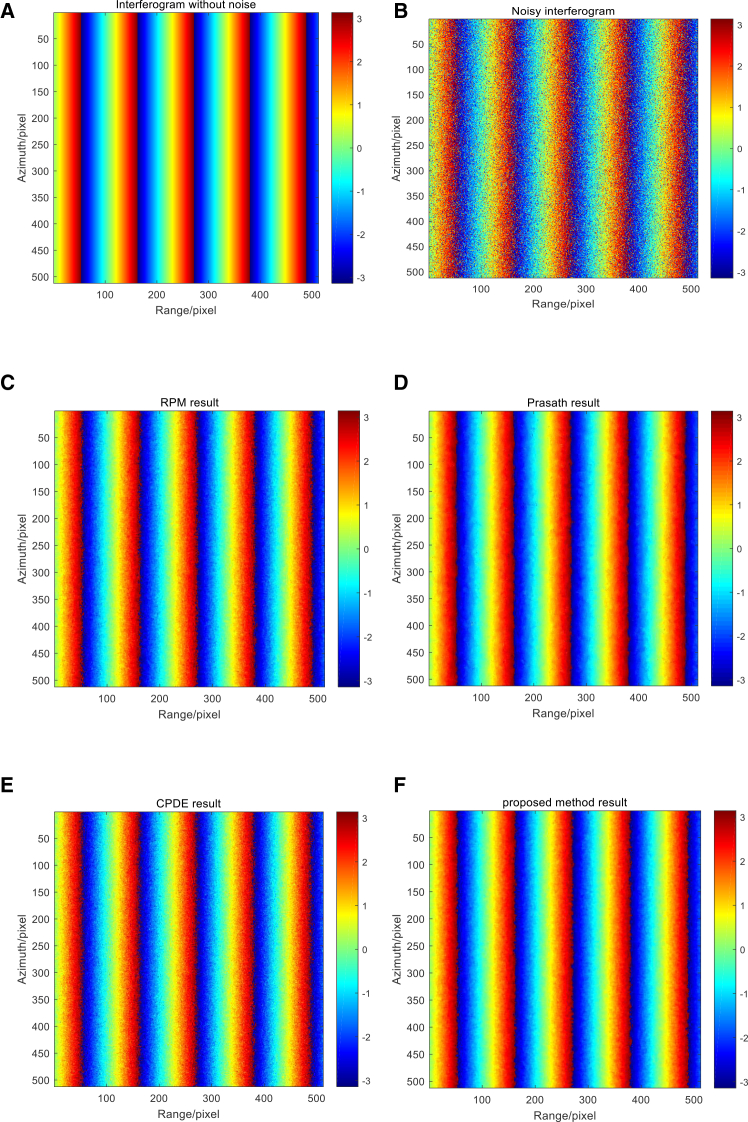
Slope terrain simulation results (A) Interferogram without noise. (B) Noisy interferogram. (C) RPM result. (D) Prasath result. (E) CPDE result. (F) Proposed method result.

**Figure 3:**
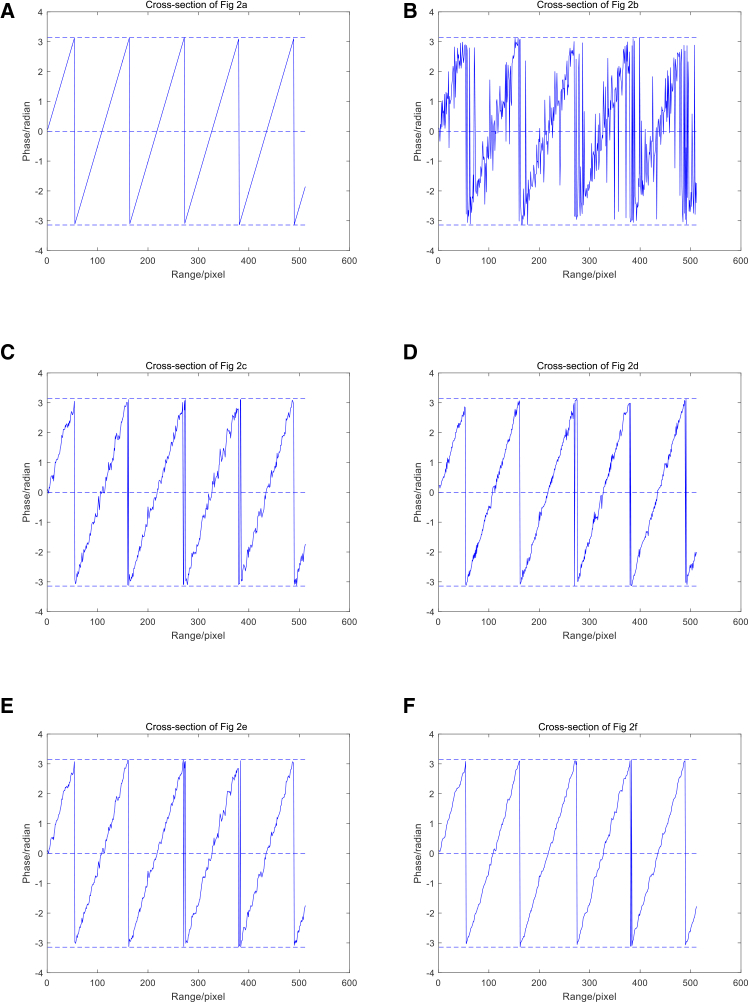
Cross-section of figure 2 (A) Interferogram without noise. (B) Noisy interferogram. (C) RPM result. (D) Prasath result. (E) CPDE result. (F) Proposed method result.

**Figure 4:**
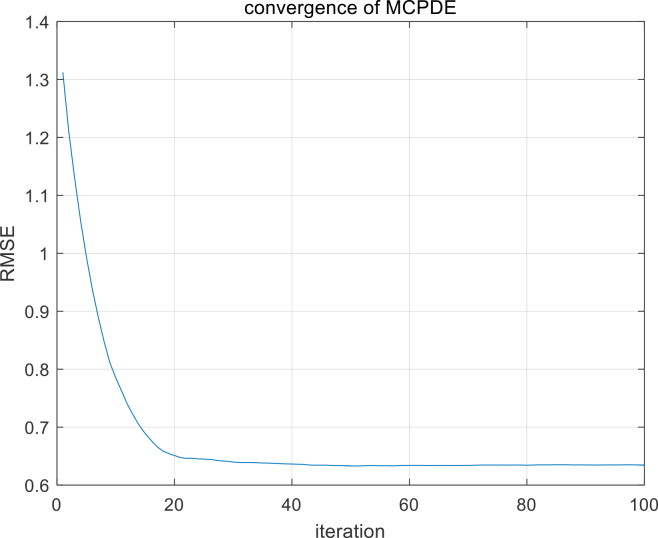
RMSE curve of iterations

A 512 × 512-pixel interferogram of a smooth slope terrain is first simulated, as shown in Figure 2A. Figure 2B shows the noisy interferogram, and Figures 2C–2F present the denoised results of the RPM, Prasath, CPDE, and proposed MCPDE, respectively. The noise in Figures 2C–2F sharply decrease than Figure 2B, which indicate that all four methods can effectively remove noise. The filtered interferograms become progressively clearer from Figures 2C–2F. Especially in the edge of fringes, it is smoother from Figures 2C–2F, which verifies that the proposed method has better performance in noise removal ability than other methods. For a direct comparison of the performance of these methods, cross-sectional profiles were extracted from Figure 2, as shown in Figure 3. In one period (from -*π* to *π*), the smoother the line, the better denoising effect. As shown in Figures 3C–3F, the proposed method yields a smoother profile than the other methods, indicating a superior denoising ability. Meanwhile, Figure 3F demonstrates that the edge of the fringe is more similar to that in Figure 3A than other methods, indicating that the introduced method has a better edge preservation ability. The quantitative evaluation criterion RMSE for each method is presented in Table 1. The data in Table 1 indicate that the proposed method achieves the best performance on edge preservation. The runtime of MCPDE is shorter than traditional CPDE method. Figure 4 gives the curve of RMSE and iteration times, which can prove the proposed method is convergent. The iteration stops when the RMSE difference between two successive iterations falls below a preset tolerance.

**Table 1:** RMSE of each method for slope terrain

	RPM	Prasath	CPDE	Proposed method
RMSE	0.737	0.703	0.69	0.63
Time(s)	7.3	6.23	6.85	5.44
EPI	0.78	0.8	0.81	0.89

**Figure 5:**
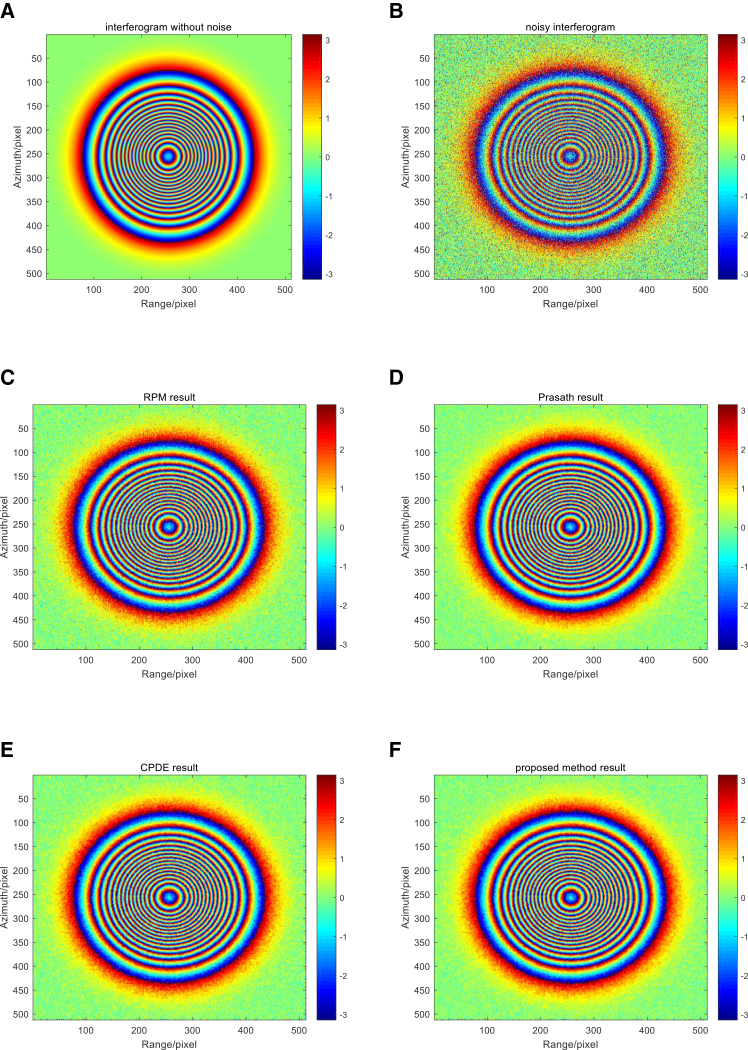
Hill terrain simulation results (A) Interferogram without noise. (B) Noisy interferogram. (C) RPM result. (D) Prasath result. (E) CPDE result. (F) Proposed method result.

**Figure 6:**
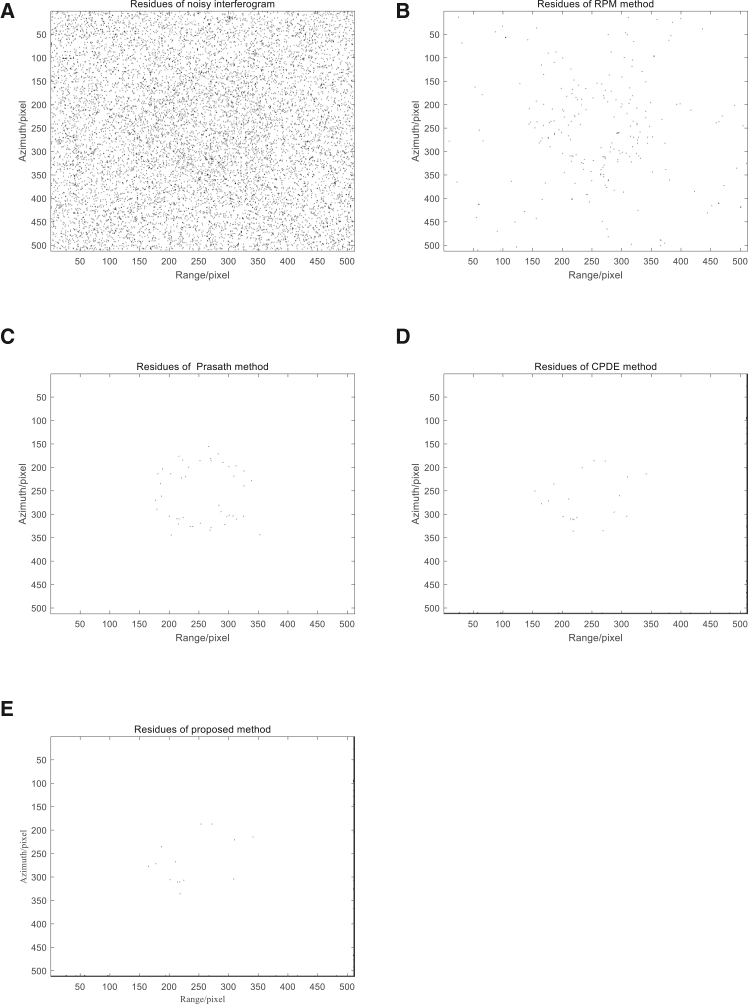
Residue distribution map for each method (A) Noisy interferogram. (B) RPM result. (C) Prasath result. (D) CPDE result. (E) Proposed method result.

**Figure 7:**
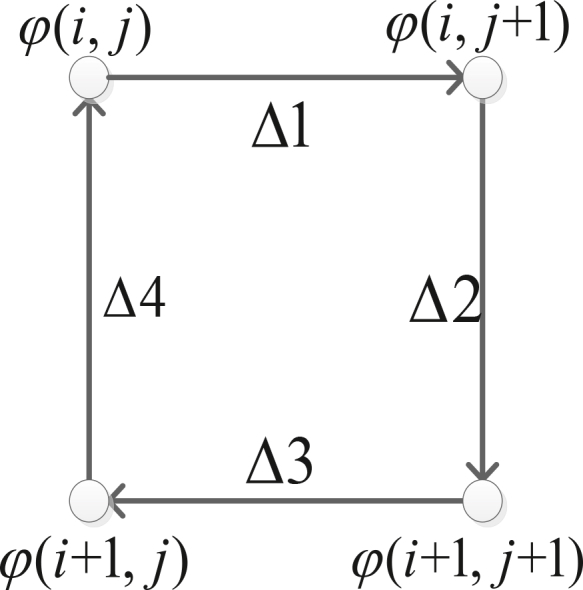
Residual point diagram

The other simulation scene is a Gaussian hill. The ideal interferogram without noise is shown in Figure 5A. The noisy interferogram is generated by adding additive noise to the interferogram in Figure 5A, resulting in Figure 5B. Figures 5C–5F present the denoised images from the RPM, Prasath, CPDE, and proposed models, respectively. Comparing Figure 5F with Figures 5C–5E, the circles in Figure 5F are clearer than Figures 5C–5E, especially in the center area where the fringes are dense. The results show that the noise removal ability of the proposed method is better than the other methods. The number of residues is an important indicator in evaluating the effectiveness of noise removal. Therefore, we present residue distribution maps for the four methods after filtering. Figure 6A shows the residue map of the noisy interferogram in Figure 5B, which has a residue count of 200,074. Figures 6B–6E show the residue maps of the RPM, Prasath, CPDE, and the proposed models, respectively. From Figure 6, it is evident that the number of residues decreases gradually across the four methods. To compare the performance quantitatively, the residue number, RMSE, and time cost for these four methods are given in Table 2. The data in Table 2 indicate that the proposed method performs better than the others. The defination of residue number can be seen in Figure 7.

**Table 2:** Comparison of the results for each method

	RPM	Prasath	CPDE	Proposed method
Residue number	508	88	79	58
RMSE	0.91	0.806	0.79	0.761
Time(s)	5.1	7.43	7.65	6.19
EPI	0.66	0.75	0.76	0.79

### Analysis of real data experimental results

**Figure 8:**
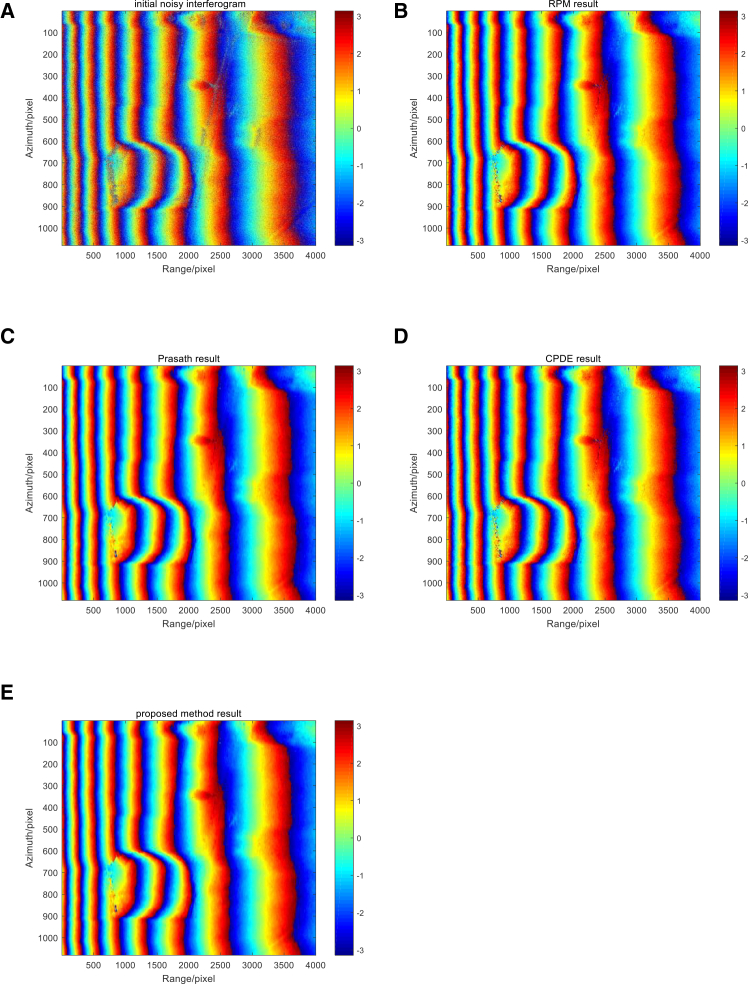
Real data processing result (A) Initial noisy interferogram. (B) RPM result. (C) Prasath result. (D) CPDE result. (E) Proposed method result.

**Figure 9:**
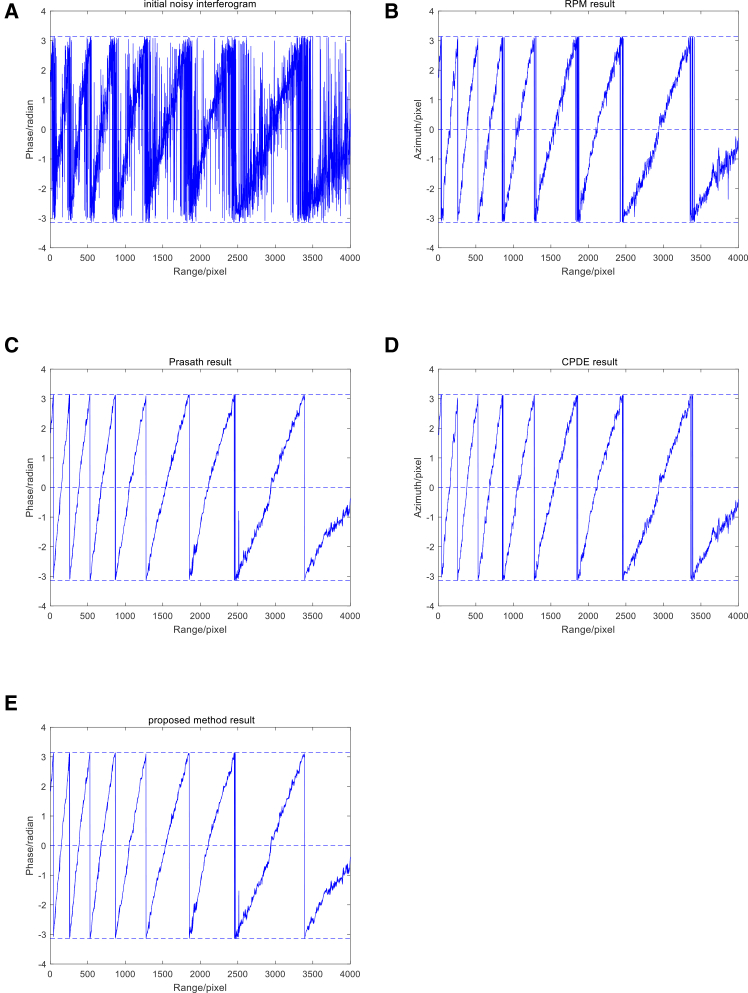
Profiles of figure 8 (A) Initial noisy interferogram. (B) RPM result. (C) Prasath result. (D) CPDE result. (E) Proposed method result.

The real InSAS data are processed by these four methods. The size of the initial interferogram with noise is 1080 × 4000, as shown in Figure 8A. Figures 8B–8E present the filtered interferograms of the RPM, Prasath, CPDE, and the proposed models, respectively. Figure 8 demonstrates that all four methods effectively filter noise. Nevertheless, a visual distinction of their differences is challenging. Therefore, we analyzed the 100th row in Figure 8 to verify their performance, as shown in Figure 9. From Figures 9B–9E, it can be seen that Figure 9E has the smoothest line among the four methods, which indicates that the introduced method outperforms the other methods in noise removal. As there are no noise-free interferograms, we cannot compare their RMSE. Thus, Table 3 presents only the residue numbers and time cost for each model. The table shows that the proposed model has the best denoising ability among the four methods. From Table 3, the proposed method has the less time cost than other CPDE method, this because the time-varying edge strength function can accelerate the convergence, which is consistent with the theoretical analysis.

**Table 3:** Comparison of results for real data processing

	Noise image	RPM	Prasath	CPDE	Proposed method
Residue number	167589	488	376	338	286
Time(s)	–	196	245	264	221

## Discussion

This article has addressed the challenge of InSAS interferogram filtering using the CPDE approach. Based on the traditional CPDE model, a modified CPDE method is proposed. In the proposed method, the time-variation feature of the edge strength function is considered and a time function is introduced to control the value of the edge fidelity term, ensuring that the image edges do not deteriorate over time. Different terrain simulation experiments and real InSAS data are processed using our method to demonstrate its effectiveness in noise removal and edge preservation. In practical engineering applications, it is not the case that the less noise there is, the better. For instance, in the flat seafloor, there is a small object, may be in the image, it just few pixels. If one merely focuses on reducing noise, this small target point might be smoothed out, thereby losing its significance. So the interferogram filtering strength should be considered with actual requirements. This approach can be used in various application scenarios, such as speckle noise removing in SAR/SAS and medical image denoising. A limitation of the proposed method is the complexity of its solution process. So, it cannot be used for real time InSAS processing. The future work is the optimization of the proposed algorithms to reduce processing time, such as parallel processing or approximation techniques, to make it feasible for real-time applications.

### Limitations of the study

The solution of CPDE equations is usually achieved through the finite difference method. However, the finite difference method has low computational efficiency and its convergence speed is highly dependent on the initial values.

## Resource availability

### Lead contact

Requests for further information and resources should be directed to and will be fulfilled by the lead contact, Pan Huang (wfxyhp@wfu.edu.cn).

### Materials availability

This study did not generate new unique reagents.

### Data and code availability

• All data are available in the supplemental information.
• All original code is available in this paper’s supplemental information.
• Any additional information required to reanalyze the data reported in this paper is available from the lead contact upon request.

## Acknowledgments

This work was supported in part by the 10.13039/501100007129Natural Science Foundation of Shandong Province (nos. ZR2023MD122 and ZR2024MA013), 10.13039/501100001809National Natural Science Foundation of China (no. 12301417) and the Weifang Science and Technology Development Program Project (no. 2023GX013).

## Author contributions

Conceptualization, P.H. and M.W.; methodology, P.H. and Y.F.; investigation, P.H. and Y.F.; writing – original draft, M.W. and L.G.; writing – review and editing, M.W. and L.G.; funding acquisition, P.H.; resources, P.H.; supervision, P.H. and Y.F.

## Declaration of interests

The authors declare that they have no known competing financial interests or personal relationships that could have appeared to influence the work reported in this paper.

## STAR★Methods

### Key resources table

**Table:** 

REAGENT or RESOURCE	SOURCE	IDENTIFIER
**Software and algorithms**		

MATLAB version R2021a	MathWorks	https://ww2.mathworks.cn

### Experimental model and study participant details

Omitted as our study does not involve biological models.

### Method details

#### Modified CPDE method

A shortcoming of Prasath model is the balancing parameter *λ* is a constant, implying that throughout the filtering process, the weights of the diffusion and fidelity terms remain fixed. The disadvantage of CPDE in is that the filtering strength and edge preservation capability do not change over time.^66^ This may lead to over-filtering and edge blurring in the edge area of the image. To overcome this problem, a modified CPDE (MCPDE) model is introduced in this paper as follows:

(Equations 6 and 7) $$\left\{ \begin{array}{l} \frac{\partial u}{\partial t}=\alpha g\left(\left|\nabla v\right|\right)\left|\nabla u\right|\nabla \cdot \left(\frac{\nabla u}{\left|\nabla u\right|}\right)+\alpha \nabla \left(g\left(\left|\nabla v\right|\right)\right)\cdot \nabla u-\beta \left(u-u_{0}\right)\\ \frac{\partial v}{\partial t}=a\left(t\right)\nabla \cdot \left(\frac{\nabla v}{\left|\nabla v\right|}\right)-b\left(t\right)\left(v-u\right) \end{array}\right.$$

In (6), *u*_0_ is the initial image, *g*(|∇*v*|) = 1/(1+|∇*v*|^2^/*K*^2^), which controls the speed of diffusion. The first term on the right-hand side of (6) is termed diffusion term, the second term is called convective term, and the third term is the fidelity term, which ensures that *u* in the filtered image and original image *u*_0_ will not differ considerably. *α* and *β* are constant, which balance effect of the diffusion term and fidelity term. Their values can be adjusted empirically. *a*(*t*) and *b*(*t*) are functions of time t. As time increases, *a*(*t*) decreases to ensure the filtering effect becomes weaker, while *b*(*t*) increases to enhance the edge preservation capability (such as *a*(*t*) = *e*^−0.2*t*^, *b*(*t*) = 0.5 + 0.01*t*). The edge function *v* modulates the edge-filtering intensity during diffusion, and *v* can be obtained by solving (7), which is derived by minimizing the following energy function^65^

(Equation 8) $$E\left(v\right)=\iint a\left(t\right)\left|\nabla v\right|+\frac{b\left(t\right)}{2}{\left|v-u\right|}^{2}dxdy$$

This approach effectively increases the consistency of *u* and *v*.

The process for solving the introduced MCPDE method is given in Figure 1. The first step is to set the initial value of *u* and *v* as the input noisy image, provide the parameters *α* and *β*, and establish the iteration termination condition. The second step is to solve (7) to obtain *v* and compute *g*(|∇*v*|) in (6). The third step is to solve (6) to get *u*. Next, check whether the stopping condition is satisfied. If it is, output the result; otherwise, return to the second step.

#### Numerical implementation

The proposed MCPDE model (6)-(7) can be implemented using various methods.^48^ This study selected a standard finite difference method.

The diffusion term in (6) can be discretized as follows:

$$\left|\nabla u\right|\nabla \cdot \left(\frac{\nabla u}{\left|\nabla u\right|}\right)=\frac{u_{x}^{2}u_{yy}-2u_{x}u_{y}u_{xy}+u_{y}^{2}u_{xx}}{u_{x}^{2}+u_{y}^{2}}$$

where *u*_*x*_ and *u*_*y*_ is the first-order partial derivative of *u* corresponding *x* and *y* directions, respectively. Similarly, *u*_*xx*_ and *u*_*yy*_ are the second-order partial derivative of *u*, and *u*_*xy*_ is the mixed partial derivative. The convective term in (6) can be represented by the difference scheme as follows:

$${\left(\nabla g\cdot \nabla u\right)}_{ij}=\max\left(\Delta_{i}g_{ij},0\right)\Delta_{i}^{-}u_{ij}+\min\left(\Delta_{i}g_{ij},0\right)\Delta_{i}^{+}u_{ij}+\max\left(\Delta_{j}g_{ij},0\right)\Delta_{j}^{-}u_{ij}+\min\left(\Delta_{j}g_{ij},0\right)\Delta_{j}^{+}u_{ij}$$

where Δ_*i*_*g*_*ij*_ is the central difference for *g* along the *x* direction, given by: Δ_*i*_*g*_*ij*_ = [*g*(*i*,*j* + 1)-*g*(*i*,*j* - 1)]/2. $\Delta_{i}^{+}u_{ij}$ and $\Delta_{i}^{-}u_{ij}$ denote forward and backward differences for *u* along x direction, respectively, which can be expressed as follows:

$$\Delta_{i}^{+}u_{ij}=u\left(i,j+1\right)-u\left(i,j\right),\Delta_{i}^{-}u_{ij}=u\left(i,j\right)-u\left(i,j-1\right)\text{.}$$

Similarly, Δ_*j*_*g*_*ij*_ denotes the central difference for *g* along the *y* direction, Δ_*j*_*g*_*ij*_ = [*g*(*i* + 1,*j*)-*g*(*i* - 1,*j*)]/2, $\Delta_{j}^{+}u_{ij}$ is the forward difference for *u* along the *y* direction, $\Delta_{j}^{+}u_{ij}=u\left(i+1,j\right)-u\left(i,j\right)$, and $\Delta_{j}^{-}u_{ij}$ represents the backward difference for *u* along the *y* direction, $\Delta_{j}^{-}u_{ij}=u\left(i,j\right)-u\left(i-1,j\right)$.

The first term in (7) is expressed as follows:

$$\nabla \cdot \left(\frac{\nabla v}{\left|\nabla v\right|}\right)=\frac{v_{x}^{2}v_{yy}-2v_{x}v_{y}v_{xy}+v_{y}^{2}v_{xx}}{{\left(v_{x}^{2}+v_{y}^{2}\right)}^{3/2}}\text{.}$$

The discretization of the first-order derivative for time *t* in (6) and (7) can be performed using a semi-implicit format, denoted as follows:

$$\frac{\partial u}{\partial t}=\frac{u_{i,j}^{n+1}-u_{i,j}^{n}}{\Delta t}\text{,}$$

where Δ*t* is the time step and *n* is the iteration times.

#### Evaluation criteria

InSAS interferogram denoising has two objectives: to filter out noise as far as possible and to maintain detailed information, such as interferometric fringes and edges. To compare these methods quantitatively, we used three commonly employed evaluation criteria.

#### Residue numbers

For InSAS interferogram denoising, the number of residual points is a crucial metric for measuring the effectiveness of denoising methods.^38^ The concept of residuals in complex field was first proposed by Goldstein.^67^ In complex field, if the function *p*(*x*) is analytic at a point *x*_0_ and the closed curve *L* is entirely within the neighborhood of *x*_0_, Cauchy’s integral theorem gives: ∮_*L*_*p*(*x*)*dx* = 0. However, when *x*_0_ is an isolated singularity, then ∮_*L*_*p*(*x*)*dx* ≠ 0. *p*(*x*) can be expanded into a Laurent series in the neighborhood of *x*_0_ and integrate term-by-term on both sides. The rest of the integrals are zero except for one term, which is 2*πC*_-1_ when *n* = −1 that is, ∮_*L*_*p*(*x*)*dx* = 2*πiC*_-1_. The value obtained by dividing this integral value by 2*πi* is called the residue of *p*(*x*) at point *x*_0_, which can be written as *R*e*s*[*p*(*x*),*x*_0_] = ∮_*L*_*p*(*x*)*dx*.

In interferogram, the calculation of residue is implemented by narrowing the integral curve to four adjacent points. That is to find the sum of the phase differences in the loop formed by these four adjacent pixels, and determine whether this sum is zero. If the sum is not zero, it is called a residue point. The detailed calculation process of residual points can be formulated as follows:

$$\left\{ \begin{array}{l} \Delta 1=W\left\{\varphi \left(i,j+1\right)-\varphi \left(i,j\right)\right\}\\ \Delta 2=W\left\{\varphi \left(i+1,j+1\right)-\varphi \left(i,j+1\right)\right\}\\ \Delta 3=W\left\{\varphi \left(i+1,j\right)-\varphi \left(i+1,j+1\right)\right\}\\ \Delta 4=W\left\{\varphi \left(i,j\right)-\varphi \left(i+1,j\right)\right\}\\ q=\Delta 1+\Delta 2+\Delta 3+\Delta 4 \end{array}\right.$$

where *φ* is the wrapped phase of the interferogram, and *W* is the mod 2*π* operation. When *q* ≠ 0, the pixel (*i*,*j*) in the top left corner is a residue point, as shown in Figure 7.

The interferogram is more severely contaminated by noise when there are more residual points in the image. However, a lower residue count is not always better, as over-filtering can reduce residues at the expense of losing detailed information (such as slender edges and small target points). Consequently, distinguishing these details could become difficult if excessive filtering is applied.

#### Root-Mean-Square error (RMSE)

The RMSE is a criterion for evaluating the deviation between the initial interferogram without noise and the denoised interferogram. It is denoted as follows:

$$RMSE=\sqrt{E\left\{{\left(\left|u_{1}\right|-\left|u_{0}\right|\right)}^{2}\right\}}$$

where *u*_0_ is the interferogram without noise and *u*_1_ is the interferogram after filtering. *E* stands for mathematical expectation. The smaller the RMSE after filtering, the better the edge-maintaining ability of the denoising method.

#### Edge preservation index (EPI)

EPI can be used to measure the degree of change in the image’s edges before and after noise removal. It is defined as follows:

$$EPI=\frac{\sum \left[\left|p_{s}\left(i,j\right)-p_{s}\left(i+1,j\right)\right|+\left|p_{s}\left(i,j\right)-p_{s}\left(i,j+1\right)\right|\right]}{\sum \left[\left|p_{0}\left(i,j\right)-p_{0}\left(i+1,j\right)\right|+\left|p_{0}\left(i,j\right)-p_{0}\left(i,j+1\right)\right|\right]}$$

where *p*_0_(*i*,*j*) and *p*_*s*_(*i*,*j*) are the phase value of the edge pixels before filter and after filter respectively. The larger the EPI, the better the edge preservation.

### Quantification and statistical analysis

All experiments are implemented in MATLAB 2021a.The simulation experiments and real trial data processing results are evaluated by three criteria: residue numbers, RMSE and EPI. All the statistical analysis results and parameters are reported in results section and Tables 1, 2, and 3.

## Supplemental information

Code S1. The code of the simulation experiment

Data S1. The simulation data of the experiment
